# Effect of various root canal irrigants on bond strength with two obturating systems

**DOI:** 10.6026/9732063002001169

**Published:** 2024-09-30

**Authors:** Hardik Kumar Babulal Patel, Vaishali Jitendra Kalburge, Nimisha Chaudhary, Payal Patel, Ekta Chaudhari Desai, Priyal V. Shah, Sindhu Soumya Dash

**Affiliations:** 1 Department of Conservative Dentistry and Endodontics, Siddhpur Dental College and Hospital, Dethali, Siddhpur - 384151, Gujarat, India; 2 Department of Conservative Dentistry and Endodontics, Private Practitioner, Anand Dental Care, Patan - 384265, Gujarat, India; 3 Kalinga Institute of Dental Sciences, Bhubaneswar, Odisha, India

**Keywords:** Bond strength, obturating systems, root canal irrigant

## Abstract

Three-dimensional obturation and careful chemo-mechanical preparation of the root canal system are essential for successful endodontic therapy. The marginal adaption and bond strength of root canal sealers to dentin are critical for the effectiveness of various endodontic operations. Hence, bond strength of Thermo-plasticized Gutta-Percha/ AH Plus sealer and ActiV GP root canal obturation system was compared following EDTA, MTAD, CHX, and Distilled Water used as a final irrigation rinse. The sample consisted of forty extracted teeth which were divided into four groups based on the final irrigation rinse (*i.e.*,) Group A 3% NaOCl followed by saline and 17% EDTA irrigation as a final rinse, Group B - 3% NaOCl solution followed by saline and MTAD irrigation as a final rinse, Group C - 3% NaOCl solution followed by saline and 2% CHX irrigation as a final rinse, Group D - 3% NaOCl solution followed by saline and distilled water irrigation as a final rinse. Each group was further subdivided based on the obturation system as; thermo-plasticized Gutta-Percha and AH Plus sealer (subgroup 1) and ActiV GP obturation system (subgroup 2). Middle and coronal thirds of every root was cut horizontally with two milli-metres thickness. After that, each slice was examined under a stereomicroscope to measure the diameter of each obturation site and snap photos of each slice's was taken at two sides using a digital camera. Later, an Instron Universal Testing Machine was used to analyze the bond strength of each specimen. CHX- Thermo-plasticized Gutta-Percha /AH Plus (3.654 + 0.056MPa) yielded the maximum average bond strength value. The minimal mean bond strength was noted for distilled water/ActiVGP (1.313 + 0.014 MPa). The other subgroups yielded intermediate bond strength values. Statistically considerable variation was found between subgroup A1 EDTA- Thermo-plasticized Gutta-Percha /AH Plus and subgroup B1 MTAD- Thermo-plasticized Gutta-Percha/AH Plus respectively. It can be found that use of 2% CHX as a final rinse considerably increases the bond strength of both Thermo-plasticized Gutta-Percha/AH Plus and ActiV GP obturation systems.

## Background:

Three-dimensional root canal obturating and comprehensive chemo-mechanical root canal system preparation are essential for successful endodontic therapy. When mechanical instrumentation is used for the dentinal tubules get clogged and an amorphous, uneven smear film covers the canal dentinal surfaces [1-2]. Existence of smear layer impairs the proper root canal sealing. Hence smear layer removal were tried with several root canal irrigants. Sodium hypochlorite (NaOCl) has long been known for its ability to dissolve tissue and remove endodontic smear layers [2]. It is poisonous at high concentrations and can harm the periapical and oral tissues. Chlorhexidine gluconate (CHX) can be considered as a substitute irrigating solution for NaOCl since CHX can precipitate and coagulate the intracellular components of bacteria and it has bactericidal properties [3]. Additionally, if it is used as an endodontic irrigant, its antibacterial action would continue in the root canal for a period of 12 weeks; nevertheless, it does not fulfil other necessary functions for an optimal irrigating solution [4]. For the purpose of eliminating the smear layer, a number of chelating chemicals have been suggested, such as RC-Prep, BioPure MTAD, Citric acid, EDTAC, REDTA, Glyde, apple cider vinegar, and EDTA. EDTA is a highly accepted chelating agent in endodontics because it efficiently eliminates the smear layer [5]. MTAD, an endodontic irrigant, has been available recently. It contains 3% Tetracycline isomer, 4.25% Citric acid, 0.5% Polysorbate and Tween 80 detergent. Research has demonstrated that MTAD is a biocompatible endodontic irrigant that is clinically beneficial and may have long-lasting antimicrobial effects [6 -7]. Under mechanical agitation, MTAD and EDTA can both totally remove the endodontic smear layer and smear plugs [8]. It is advised to use this irrigant as a last rinse following an initial irrigation with 3% NaOCl. In both static and dynamic scenarios, the integrity of the seal in a root canal filling depends on the bond strength of the root canal sealers to dentine [9]. The ideal endodontic cement should stick to the canal wall and gutta-percha core, thus sealing the root canal gap. The AH plus sealer (Dentsply, Detrey, Gmbh, Germany) is an example of an epoxy resin-based sealer cement that is extensively used due to its good physical qualities, reduced solubility, ability to apically seal, micro retention to root dentin and adequate biological performance[10]. In the field of endodontics, adhesive dentistry has recently been launched by manufacturers. A novel glass-ionomer root canal filling method called ActiV GP (Brasseler Savannah, GA, USA) has been introduced to the market with the goal of producing a single-cone mono-block obturation. The system consists of gutta-percha cones coated and impregnated with glass ionomer, which can be bonded to a sealing agent made of polyacrylic acid and powdered barium aluminosilicate glass [11]. Only few researches have been done on how various irrigants affect the strength of the sealer-dentin bond when utilizing the ActiV GP obturation system [12]. Therefore, it is of interest to assess the push-out bond strength of two distinct obturating systems using irrigating solutions containing EDTA, MTAD, CHX and distilled water.

## Materials and Methods:

**Figure 1:**
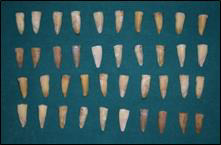
Tooth samples used for the study

**Figure 2:**
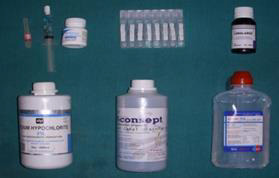
Irrigants used in the study

**Figure 3:**
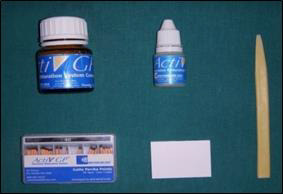
ActiV GP obturation system

**Figure 4:**
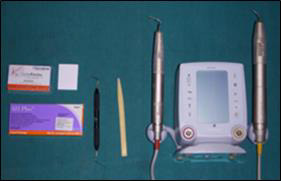
Thermo-plasticized Gutta-Percha/ AH Plus obturation system

**Figure 5:**
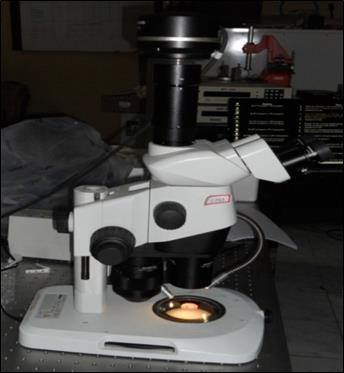
Stereomicroscope

**Figure 6:**
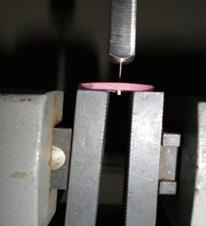
Application of compressive load

The current in vitro research was done in the Conservative Dentistry and Endodontics Department. The study was done on single rooted human mandibular premolar teeth which were recently extracted for periodontal or orthodontic reasons. Teeth with any pathology, with immature apex, accessory canals were eliminated from this research. The collected 40 teeth were cleaned, sterilized and then decoronated using a high-speed carbide bur and water spray to obtain approximately 14-mm long root segments (Figure 1). Canal space of all the prepared specimens were instrumented using Ni-Ti rotary instruments by crown down approach. These specimens were then alienated into 4 groups based on the final irrigation rinse with 10 samples in each group (Figure 2); (*i.e.*,) Group A - 5 ml of 3% NaOCl solution was used for 1 min, subsequently irrigation with 5 ml of Saline and 17% EDTA as a final rinse. Group B -5 ml of 3% NaOCl solution was used for 1 min, subsequently irrigation with 5 ml of Saline and MTAD as a final rinse. Group C - 5 ml of 3% NaOCl solution was used for 1 min, subsequently irrigation with 5 ml of Saline and 2% CHX as a final rinse. Group D - 5 ml of 3% NaOCl solution was used for 1 min, subsequently irrigation with 5 ml of Saline and Distilled Water as a final rinse. Each group was further subdivided into 2 subgroups based on the obturation system used. In the first subgroup, root canals were obturated using Thermo-plasticized Gutta-Percha and AH Plus sealer, and in second subgroup, the root canals were filled using the ActiV GP obturation system. Sub groups was done based on obturation material with irrigants as; subgroups A1 (EDTA/ Thermo-plasticized Gutta-percha - AH Plus), A2 (EDTA/ActiV GP) (Figure 3), B1 (MTAD/ Thermo-plasticized Gutta-percha-AH Plus) (Figure 4) and B2 (MTAD/ActiV GP), C1 (CHX/ Thermo-plasticized Gutta-percha-AH Plus) and C2 (CHX/ActiV GP), D1 (Distilled Water/ Thermo-plasticized Gutta-percha - AH Plus) and D2 (Distilled Water/ActiV GP).This technique was employed for Subgroup as; A2 (NaOCI/EDTA - ActiV GP),B2 (NaOCI/MTAD - ActiV GP), C2 (NaOCI/CHX - ActiV GP) & D2 (NaOCI/ Distilled Water - ActiV GP). After the teeth were obturated, they were implanted in an auto-polymerizing acrylic resin. Next, utilizing a water-cooled precision saw (Micracut, Metkon, Bursa, Turkey), three horizontal portions of 2 mm thickness were cut from the coronal and middle third. The slices were maintained wet in a saline-filled container. After that, each slice was placed in a stereomicroscope (Figure 5) (Lawrence & Mayo, London) with a 3x magnification, and each obturation site's diameter was measured. Every slice's thickness was determined using a computerized calliper (Aerospace, China). Later each specimen was subjected to bond strength analysis using an Instron Universal Testing Machine (Zwick/Roell Instruments Ltd.UK) (Figure 6). The obtained data was statistically evaluated using SPSS software version 23.0 using student 't' test and one way ANOVA P<0.05.

## Results:

Group C (CHX) yielded appreciably maximum average push-out bond strength and Group D (Distilled Water) with appreciably lower average push-out bond strength. The remaining groups discovered intermediate average values of push-out bond strength. The results obtained were statistically significant (p value 0.000) (Table 1). The Mean Bond Strength between Thermo-plasticized Gutta-Percha/AH Plus sealer obturation system was statistically considerable (p value 0.000). Higher value was fond in group C1 and lowest with group D1 (Table 2).The Mean Bond Strength between Thermo-plasticized Gutta-Percha/AH Plus sealer obturation system which was statistically considerable (p value 0.000). Higher value found with group C2 and lowest with group D2 (Table 3).

## Discussion:

The marginal adaption and bond strength of root canal sealers to dentin are critical for the effectiveness of various endodontic operations and for preserving the integrity of the seal in the root canal filling [9]. Evaluating bond strength has grown in popularity as a way to assess how well dental materials adhere to dentin in root canals. Our study evaluated push-out bond strength because it is regarded as an effective and dependable technique that enables evaluation of regional variations in bond strength among root levels and bond strength of root canal sealers at adhesion interface [13]. A perfect endodontic sealer should stick strongly to Gutta-Percha as well as dentin [14]. The chemical and structural makeup of human dentin may change as a result of dentin surface treatment with various irrigation schedules, which may also have an impact on the adherence of materials to dentin surfaces [15]. A gap may grow between the sealer core and sealer dentin if the bond between them is insufficient to withstand crippling forces, which would ultimately cause the bond to collapse [16].

The AH Series (Dentsply Maillefer, Tulsa, OK) is the most commonly used resin- based sealer system, with AH Plus being the newer of the series is used with Gutta-Percha core material in our study. ActiV GP bonds chemically to tooth structure (a diffusion-based adhesion) by ion exchange between the glass-ionomer and the tooth surface. Inadequate bonding between GI and Gutta-Percha is a disadvantage with GI-based sealers. The varied irrigation regimens used in our investigation were shown to have distinct effects on the bond strength of the investigated obturation systems. There could be several reasons for the increased bond strength that was achieved with the CHX group for both obturation systems. Chlorhexidine, because of its substantive antimicrobial properties, has been recognized as an effective irrigating solution. CHX is a cationic bis-guanide which gets adsorbed to root dentin and released as long as 48 to 78 hrs [3]. The presence of oil based materials in AH Plus composition prevent the complete wetting of root canal walls and adhere poorly to humid dentin resulting in poor adaptation of the material to the canal walls, and this effect is nullified by property of CHX [10]. By improving the dentin surface's cationic charging, CHX increases the polycarboxylic group of the glass-ionomer's reaction. Our results are in agreement with a study done by Ahmed *et al.* results indicated highest mean bond strength value for CHX/ActiV GP and accomplished that bonding strength of Acti-V GP can be improved by final irrigation with 2% CHX [17]. Our findings are also congruent with Erdemir *et al.* study, in which teeth treated with chlorhexidine solution showed the maximum bond strength readings [18]. Most people agree that EDTA is the best chelating agent because of its strong lubricating qualities. It reduces the dentinal wall's capacity to absorb water [19]. Additionally, it aids in the smear layer's elimination [20]. As a potent antioxidant, EDTA facilitates full resin poly-merisation and strengthens the resin-dentin connection [12]. Our findings are in association with a study by Sly *et al.* that found that the novel obturation technique has a lower push-out binding strength to intra-radicular dentin than Gutta-Percha/AH Plus, Epiphany/Resilon [21]. In our investigation, MTAD using the ActiV GP obturation system produced lower bond strength than MTAD with Thermo-plasticized Gutta-Percha/AH Plus obturation system. The different characteristics of MTAD may be to reason for this. The glass-ionomer based sealer adhered well to the dentinal surface treated by the final watering with MTAD per se in subgroup B2 (MTAD/ActiV GP). When utilizing MTAD as a final irrigant, the values of push-out bond strength recorded higher with gutta-percha/AH plus coincide with those reported by De-Dues *et al.* [22]. Our results indicated that thermo-plasticized Gutta-Percha /AH Plus obturation system showed better bond strength when EDTA, MTAD, CHX and Distilled Water were used as a final rinse. Hashem *et al.* concluded that the bond strength of gutta-percha/AH plus was adversely affected by MTAD and MTAD/CHX [17]. In our study ActiV GP obturation system recorded intermediate bond strength this could be due to, non - homogenous coating of Glass-ionomer filler on the surface of ActiV GP/GI sealer cones [23]. Though NaOCl is one of the most commonly used endodontic irrigating solution and it possesses excellent antimicrobial and tissue dissolving abilities. CHX and NaOCl exhibit comparable antibacterial action against common organisms isolated from the root canal system, according to research by Vianna *et al.*. In the present study it can be said that CHX used as a final irrigant did not influence the bond strength of the different obturating systems. The use of EDTA and MTAD as a final irrigant also performed better then Distilled Water.

## Conclusion:

Thermo-plasticized Gutta-Percha/AH Plus and ActiV GP obturation system bond strength was found to be greatly boosted by using 2% CHX as a final rinse. Of the two obturation systems, thermo-plasticized Gutta-Percha/AH Plus had the highest mean bond strength.

## Figures and Tables

**Table 1:** Comparison of mean bond strength between different irrigants

**Irrigant type**	**Mean Bond Strength**	**SD**	**f' value**	**p' value**
Group-A (EDTA)	2.075	0.06	254.453	0
Group-B (MTAD)	2.409	0.14		
Group-C (CHX)	3.442	0.23		
Group-D (Distilled Water)	1.483	0.18		

**Table 2:** Association of bond strength of Thermo-plasticized Gutta-Percha/AH Plus obturation system between the groups

**Sub Groups**	**N**	**Mean Bond Strength**	**SD**	**f' value**	**p' value**
Sub Group A1	5	0.016	2.131	3669.16	0
Sub Group B1	5	0.015	2.538		
Sub Group C1	5	0.056	3.654		
Sub Group D1	5	0.015	1.653		

**Table 3:** Comparison of bond strength of ActiV GP Obturation system between the groups

**Group**		**N**	**Mean Bond Strength**	**SD**	**Min**	**Max.**	**f' value**	**p' value**
A	Sub Group A2	5	2.019	0.02	2	2.05		
B	Sub Group B2	5	2.281	0.02	2.26	2.31	10003	0
C	Sub Group C2	5	3.231	0.02	3.21	3.26		
D	Sub Group D2	5	1.313	0.01	1.3	1.34		

## References

[1] Gusiyska A, Elena D (2023). Appl. Sci..

[2] Hiilsmann M (2003). Int Endod J..

[3] Wang CS (2007). J Endod..

[4] Qutieshat A (2023). The Open Dentistry Journal..

[5] Kaya S (2011). Int Dent Res.

[6] Zhang W (2003). J Endod..

[7] Torabinejad M (2003). J Endod..

[8] Mohammadi Z (2019). J Contemp Dent Pract..

[9] Moinuddin M.K (2019). Contemp Clin Dent..

[10] Zemner O (1997). Int Endod J..

[11] Donadio M (2008). Oral Surg Oral Med Oral Pathol Oral Radiol Endod..

[12] Dinesh K (2014). J Contemp Dent Pract..

[13] Goracci C (2004). Eur J Oral Sci..

[14] Vujaskovic M, Teodorovic N (2010). SrpArhCelokLek..

[15] Monticelli F (2007). Int Endod J..

[16] Diemer F (2006). J Endod..

[17] Hashem A.A.R (2009). J Endod..

[18] Ari H (2003). J Endod..

[19] Gomes B.P.F.A (2023). Braz Dent J..

[20] Dogan Buzoglu H (2007). Int EndodJ..

[21] Sly M.M (2007). J Endod..

[22] De-Deus G (2008). J Endod..

[23] Vianna M.E (2004). OOOE..

